# Predicting copper-, iron-, and zinc-binding proteins in pathogenic species of the *Paracoccidioides* genus

**DOI:** 10.3389/fmicb.2014.00761

**Published:** 2015-01-09

**Authors:** Gabriel B. Tristão, Leandro do Prado Assunção, Luiz Paulo A. dos Santos, Clayton L. Borges, Mirelle Garcia Silva-Bailão, Célia M. de Almeida Soares, Gabriele Cavallaro, Alexandre M. Bailão

**Affiliations:** ^1^Biochemistry and Molecular Biology, Laboratório de Biologia Molecular, Universidade Federal de GoiásGoiânia, Brazil; ^2^Magnetic Resonance Center, University of FlorenceSesto Fiorentino, Italy

**Keywords:** metalloproteome, bioinformatics, Paracoccidioidomycosis, metal homeostasis, virulence

## Abstract

Approximately one-third of all proteins have been estimated to contain at least one metal cofactor, and these proteins are referred to as metalloproteins. These represent one of the most diverse classes of proteins, containing metal ions that bind to specific sites to perform catalytic, regulatory and structural functions. Bioinformatic tools have been developed to predict metalloproteins encoded by an organism based only on its genome sequence. Its function and the type of metal binder can also be predicted via a bioinformatics approach. *Paracoccidioides* complex includes termodimorphic pathogenic fungi that are found as saprobic mycelia in the environment and as yeast, the parasitic form, in host tissues. They are the etiologic agents of Paracoccidioidomycosis, a prevalent systemic mycosis in Latin America. Many metalloproteins are important for the virulence of several pathogenic microorganisms. Accordingly, the present work aimed to predict the copper, iron and zinc proteins encoded by the genomes of three phylogenetic species of *Paracoccidioides* (*Pb*01, *Pb*03, and *Pb*18). The metalloproteins were identified using bioinformatics approaches based on structure, annotation and domains. Cu-, Fe-, and Zn-binding proteins represent 7% of the total proteins encoded by *Paracoccidioides* spp. genomes. Zinc proteins were the most abundant metalloproteins, representing 5.7% of the fungus proteome, whereas copper and iron proteins represent 0.3 and 1.2%, respectively. Functional classification revealed that metalloproteins are related to many cellular processes. Furthermore, it was observed that many of these metalloproteins serve as virulence factors in the biology of the fungus. Thus, it is concluded that the Cu, Fe, and Zn metalloproteomes of the *Paracoccidioides* spp. are of the utmost importance for the biology and virulence of these particular human pathogens.

## Introduction

Metal ions such as copper, iron, and zinc and others play an essential role in living organisms primarily by virtue of their association with proteins, which are referred to as metalloproteins (Frausto Da Silva and Williams, 1991; Bertini et al., 2007; Festa and Thiele, 2011). Approximately one-third of all proteins studied are associated with a metal ion (Shi and Chance, 2008) whose presence is most commonly needed for the catalytic mechanism of enzymes and/or the stabilization of the tertiary or quaternary structure of proteins (Andreini et al., 2006a).

Many metals play important roles in organisms as a free ion or coupled to proteins. However, the function of Cu, Fe, and Zn is primarily related to metalloprotein dependence on those elements. Copper essentiality retains both its activity in structural stabilization and its redox ability, which is used by metalloenzymes that catalyze electron transfer reactions (Festa and Thiele, 2012). In this respect, this metal functions in a broad range of metabolic activities including, for example, energy production, iron acquisition, melanin production and antioxidant defense (Kim et al., 2008). Iron is also a redox-active element and is essential as a cofactor in the form of heme and iron-sulfur clusters in a variety of cellular processes such as respiration, amino acid metabolism, biosynthesis of sterols and DNA, peroxide detoxification, and DNA replication (Nevitt, 2011; Schrettl and Haas, 2011; Netz et al., 2012). Zinc constitutes the catalytic and/or structural core of many proteins involved, among other functions, in transcriptional control, reactive oxygen species (ROS) detoxification, carbohydrate oxidation and alcoholic fermentation (Murakami and Hirano, 2008; Wilson et al., 2012).

Although metals are fundamental for the correct functioning of cells, their excess is toxic. Thus, metal availability is tightly controlled (Valko et al., 2005; Bleackley and Macgillivray, 2011). During infection there is a battle for micronutrients where a host can either decrease metal availability to the invader or increase the metal concentration to toxic levels, and pathogens must keep metal homeostasis in host tissues to promote a successful infection (Ammendola et al., 2007; Samanovic et al., 2012; Wilson et al., 2012; Cassat and Skaar, 2013). A prerequisite to understanding the homeostatic mechanisms that maintain constant levels of the essential metals and removing the unwanted metals in an organism is the knowledge, as complete as possible, of the metalloproteins encoded by that organism. With the advent of genome sequencing, the entire proteome of several species has become available. For the majority of these data, however, there is no functional information available, as a thorough functional characterization of whole proteomes is not yet routinely possible. Thus, a systematic approach to search metalloproteins in protein sequence databases has been developed (Andreini et al., 2004) and used in a systematic description of copper, iron and zinc proteins through the three domains of life (Andreini et al., 2006b, 2007, 2008).

Paracoccidioidomycosis is a systemic mycosis restricted to Latin American countries. This disease is caused by the fungi of the species complex *Paracoccidioides* spp. The complex is composed of two species: *Paracoccidioides lutzii* and *Paracoccidioides brasiliensis*; the latter has four phylogenetic species, S1, PS2, PS3, and PS4, with a different geographic distribution (Matute et al., 2006a,b; Carrero et al., 2008; Teixeira et al., 2009, 2013; Bocca et al., 2013). *Paracoccidioides* spp. are thermodimorphic fungi that present as mycelium in the environment at temperatures of 18–25°C and as yeast in mammalian hosts at 36°C. Metal homeostasis has been well described as a determinant factor in fungal pathogenesis (Bailao et al., 2006; Schrettl and Haas, 2011; Festa and Thiele, 2012; Schneider Rde et al., 2012; Wilson et al., 2012), and *Paracoccidioides* spp. presents several metal homeostasis genes that encode for molecules that have been described as important virulence factors in fungi (Silva et al., 2011). In the present work, we used the abovementioned bioinformatics approach to predict the Cu-, Fe-, and Zn-binding proteins encoded by the genomes of the genus *Paracoccidioides*, with the aim of advancing our comprehension of the fungal metal homeostasis and virulence.

## Materials and methods

### Identification of Cu-, Fe-, and Zn-binding proteins in the *Paracoccidioides* spp. genome

Metalloproteins were identified by using the RDGB tool (Andreini et al., 2011) with default options. In the RDGB strategy, the protein domains defined in the Pfam library are used to identify putative homologs in any desired genome or list of genomes. Copper-, iron-, and zinc-binding Pfam domains were initially identified in the sequence of copper-, iron-, and zinc-binding proteins of known 3D structures, which are available from the Protein Data Bank (PDB). When a particular metal is present within the 3D structure of the protein, this information can be readily extracted from the PDB database along with the pattern of amino acids that are involved in the interaction of the protein with the metal.

The latter is referred to as the ligand binding pattern (LBP) and is defined by the identity and spacing of the amino acids, e.g., CX(4)CX(2)H, where X is any amino acid. This pattern is usefully applied as a filter to reduce the number of false positives (i.e., of the proteins predicted to bind the metal, which in reality are unable to bind it) by rejecting the proteins that lack the LBP. The LBP filter is applied by imposing that the predicted protein contains all of the ligands of the LBP with a spacing in sequence that it is maintained within ±20% (or ±1 amino acid for short spacing). The lists of copper-, iron-, and zinc-binding Pfam domains were manually refined before being used in the RDGB protocol by (I) removing the domains that did not bind the metal(s) physiologically, and (II) adding domains that were known to bind the metal(s) physiologically, although no 3D structure was available. The latter refinement is based on the annotation present in the Pfam database, which is sufficiently detailed to allow users to evaluate the actual relevance of a domain to the biochemistry under investigation (in this case, the metal-binding properties). In further detail, for each domain in the list, the annotation is examined to confirm that the domain physiologically binds that metal. If this is not the case (e.g., due to adventitious binding during crystallization or purification procedures), then the domain is rejected. When the Pfam annotation alone is not sufficient to establish whether a metal is physiologically bound, the relevant literature is analyzed. Additionally, the Pfam is queried for those domains whose annotation contains the name and/or the symbol of the metal. This typically provides additional domains that lack structural characterization but have been identified as binding a specific metal. Again, the relevant literature is also investigated.

As reported in the original RDGB paper (Andreini et al., 2011), the RDGB performance parameters are evaluated as follows: sensitivity [TP/(TP + FN)] = 97.7%; specificity [TN/(TN + FP)] = 78.8%; precision [TP/(TP + FP)] = 85.9%; and accuracy [(TP + TN)/(TP + TN + FP + FN)] = 89.6%.

### Functional classification, localization and comparisons of Cu-, Fe-, and Zn-binding proteins from *Paracoccidioides* spp.

The predicted metalloproteins of *P. lutzii Pb*01 and *P. brasiliensis Pb*18 and *Pb*03 were functionally classified with the FunCat2 scheme accessed on the Pedant database (http://pedant.gsf.de/) (Walter et al., 2009). The WolfPsort system was used to predict the putative cell localization of the proteins. The homology comparison among the metalloproteomes of the three species was performed using the BLAST tool. The metalloproteins of one phylogenetic species were compared to the corresponding metalloproteome of the two other species to find the orthologs. The structural analysis was performed to ascertain that the bound metal was completed with the I-Tasser algorithm (http://zhanglab.ccmb.med.umich.edu/I-TASSER/). BLAST and I-Tasser were used in the analysis of the species-exclusive metalloproteins. When the BLAST comparison of the metalloprotein databank results indicated a specific protein, the sequence was compared against the whole proteome and the structure of the protein was predicted. To study the potential functional associations among the copper proteins having representatives in all *Paracoccidioides* spp., a COG identification number was associated with these proteins. The list of COG identification numbers was then used to query the STRING database (Von Mering et al., 2003).

### Culture conditions and expression analysis by qRT-PCR

Yeast cells of *P. lutzii* were maintained in a solid Fava Neto's medium at 37°C. The yeasts were grown in a liquid medium for 72 h at 37°C. The cells were harvested by centrifugation and washed twice with a phosphate saline buffer and then transferred to the chemically defined medium MMcM (Restrepo and Jimenez, 1980). The iron limiting condition was generated without the addition of iron sources and the supplementation of the iron chelator BPS (bathophenanthroline-disulfonic acid) at 50 μM. The iron excess condition was induced by adding ammonium ferrous sulfate at 50 and 100 μM. Control cells were incubated in a MMcM medium with 3.5 μM of iron. The RNA extraction and quantitative RT-PCR was performed as previously described (Silva-Bailao et al., 2014). The oligonucleotides used in the PCR amplification were: Ca-transporter FW 5′- GTCGAACGAGGCAATGAGAGAG-3′; Ca-transporter RV 5′- TTGTAAAATCTTGCGCTTCGGG-3′; α-tubulin FW 5′-ACAGTGCTTGGGAACTATACC-3′; and α-tubulin RV 5′-GGGACATATTTGCCACTGCC-3′.

## Results

### Identification of copper-, iron-, and zinc-binding proteins in *Paracoccidioides* spp. genomes

The genomes of three phylogenetic species of the *Paracoccidioides* spp. complex were analyzed for Cu, Fe, and Zn proteins using two complementary approaches: all of the metal binding patterns (MBP) were retrieved from the PDB and used along with sequence analysis to scan the proteins; additionally, a library of metal-binding protein domains based on multiple sequence alignments of known metalloproteins was used (taken from Pfam) to browse the predicted proteome (Andreini et al., 2006a). In total, 25,753 proteins from the three completely predicted proteomes were subjected to analysis and 1952 metalloproteins were detected. The results also show that those proteins represent, on average, 7.6% of the predicted proteome of these fungi (Table 1).

**Table 1:** **Number of metalloproteins identified in the *Paracoccidioides* spp. genome**.

***Paracoccidioides* sp.**	**Predicted proteome**	**Cu proteins (%)**	**Fe proteins (%)**	**Zn proteins (%)**
*P. lutzii (Pb*01)	9136	26 (0.28)	115 (1.25)	522 (5.71)
*Pb*18	8741	25 (0.28)	115 (1.31)	511 (5.84)
*Pb*03	7876	26 (0.33)	111 (1.40)	501 (6.38)

The most abundant metalloproteins were zinc-binding followed by iron-binding and copper-binding (Supplementary Tables 1–3). A higher frequency of Zn-binding proteins was expected because zinc is one of the most abundant metal ions in living organisms, playing two major roles: catalytic and structural. Some of the identified metalloproteins presented ambiguity between their annotation and the identified metal domain (e.g., proteins PAAG_06410, PADG_01717, PAAG_03944, and PAAG_02157). These molecules were subjected to structural analysis and 70% were found to have the same metal (Supplementary Table 4), whereas 30% presented other putative metals, such as Mn and Ca. Some proteins presented two possible ligand metals. The most frequent combinations were Fe/Zn followed by Cu/Zn and Cu/Fe (Table 2). To investigate the results of the metalloproteins with two bound metals, the protein structure was predicted using the I-Tasser software. This approach revealed that 11.9% (Pb01), 17.5% (Pb03) and 13.5% (Pb18) of the proteins present motifs for two metals and most of them corroborate with the MBP-based prediction. The remaining metalloproteins presented only one ligand metal, and 62.1 to 81.8% of the structures were in consonance with the results based on the MBP and Pfam domains (Supplementary Table 4). The list of the metalloproteins identified in this work is available at http://www.broadinstitute.org/annotation/genome/paracoccidioides_brasiliensis/MultiHome.html.

**Table 2:** **Number of proteins with two metal-binding domains**.

***Paracoccidioides* spp**.	**Cu × Fe**	**Cu × Zn**	**Fe × Zn**
*P. lutzii*	2	6	34
*Pb18*	2	5	30
*Pb03*	2	7	31

### Copper proteins

**Figure 1:**
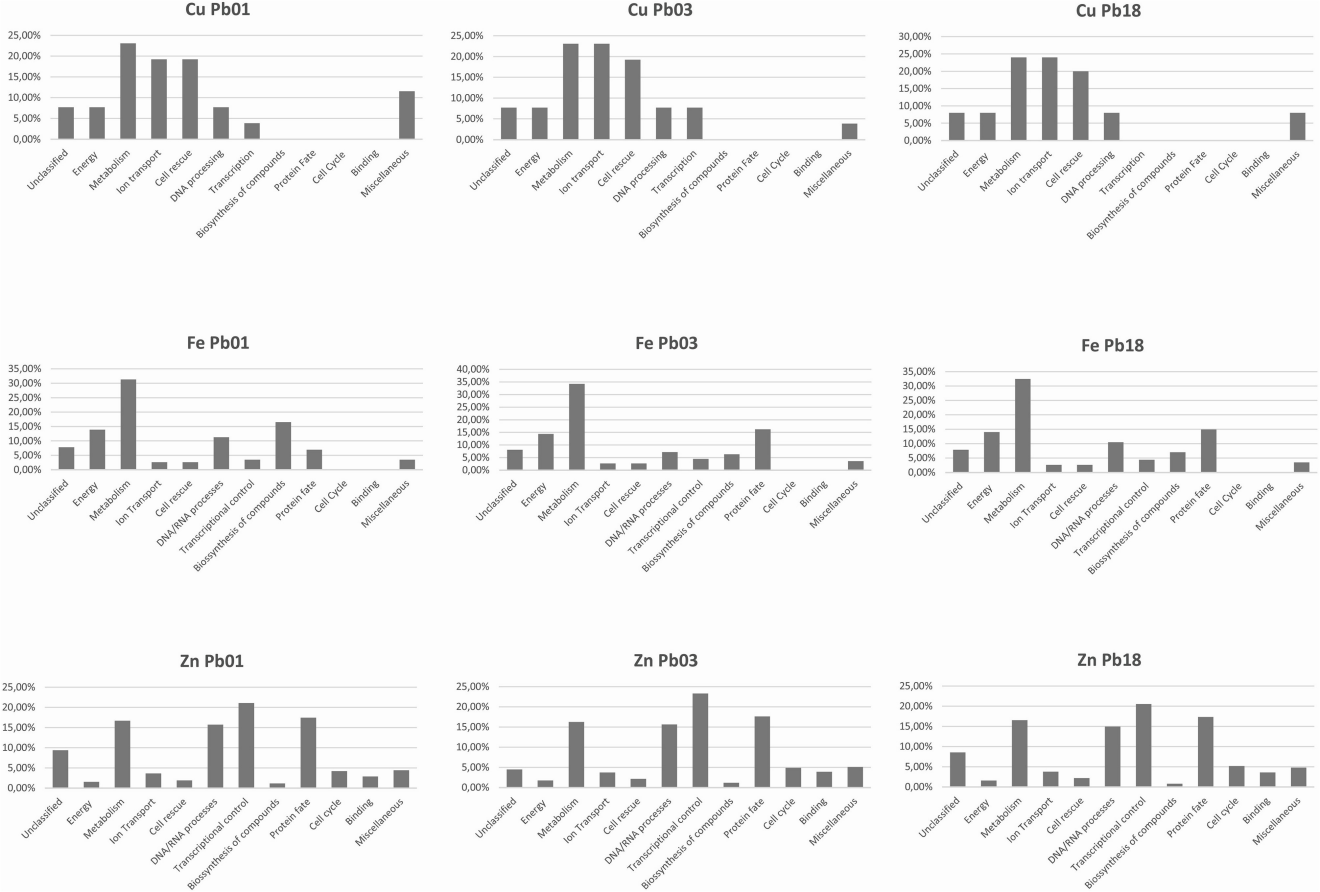
**Functional classification of Cu-, Fe-, and Zn- proteins encoded by *Paracoccidioides* spp. genomes**. The metalloproteins were classified following the FunCat2 scheme available at the Pedant database.

**Figure 2:**
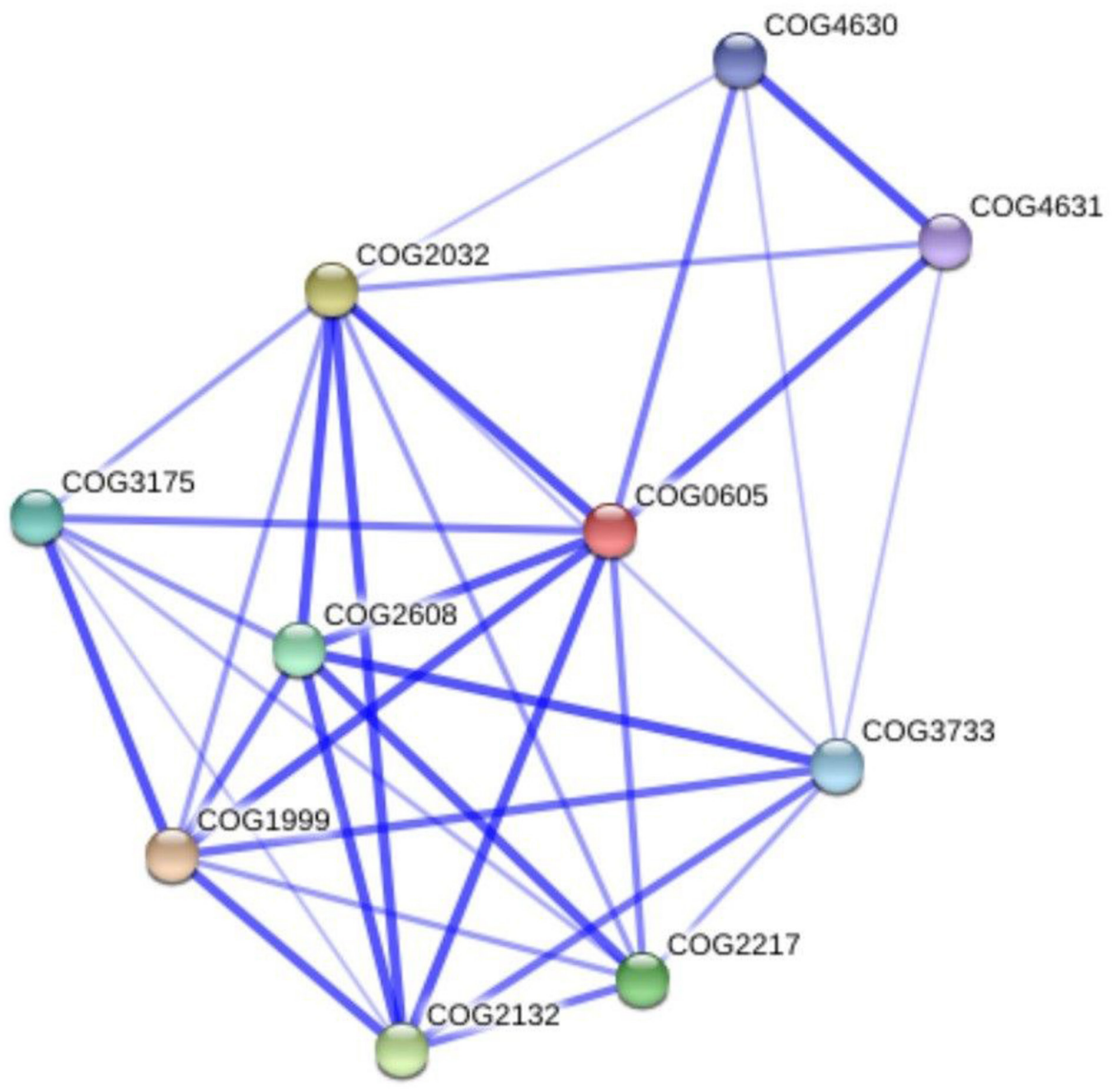
**Functional interaction graph of copper proteins from *P. lutzii* predicted proteome**. The graph was constructed in the STRING tools using standard parameters. Cu-proteins found in the present study: COG3733 copper amine oxidase; COG2608 copper-transporting P-type ATPase; COG2217 copper-transporting ATPase; COG1999 mitochondrial metallochaperone Sco1; COG2132 laccase IV; COG2032 superoxide dismutase; COG0605 cytosolic Cu/Zn superoxide dismutase; COG4631 xanthine dehydrogenase; COG4630 xanthine dehydrogenase; COG3175 cytochrome c oxidase assembly protein ctaG.

The bioinformatics analysis showed that 26 Cu-binding proteins were found in the *P. lutzii* and Pb03 genomes, whereas 25 were found in the Pb18 genome. Functional classification of the Cu proteins revealed that ion transport and melanin synthesis are the most enriched functional categories because 6 copper transporters and 3 laccases were found (Figure 1). Not surprisingly, the high-affinity copper transporter whose expression is induced in infection-like conditions (Bailao et al., 2006; Costa et al., 2007) belongs to the predicted copper proteome. Although only two proteins related to stress response were found, it is important to highlight that the two superoxide dismutases are copper dependent. Additionally, three amino oxidases contain a Cu domain, suggesting the relevance of this metal for their catalysis. *In silico* analysis also showed that 5 hypothetical proteins (Supplementary Table 1) present a Cu-binding domain. The copper proteins present in the three PS were analyzed in search of potential functional associations. To accomplish this, a COG identification number was associated with each ortholog group. According to the STRING database, 11 of 19 copper proteins are interconnected by high functional associations (score = 0.99) as shown in Figure 2. The interaction network represents a combination of different pieces of evidence that there are relationships of a functional nature among the proteins. In fact, the linkages between the pairs reveals that Cu-chaperone and Cu-transporting ATPase are core proteins that are functionally essential to metal homeostasis because the former binds to and distributes copper in the cell and the latter transports and keeps copper level in the Golgi apparatus for copper proteins such as laccases. Additionally, cu-superoxide dismutase and laccase interact with many cu-binding molecules, suggesting their participation in Cu homeostasis (Figure 2).

The cell localization prediction analysis corroborates the functional analysis and annotation of Cu-proteins (Supplementary Tables 1–3). The results indicate a high frequency of such proteins at the membranes as a result of the high numbers of copper transporters. The number of secreted proteins was also high. Among the molecules predicted to reach the extracellular environment are laccases, SOD, amine oxidase, polyphenoloxidase, and tyrosinase. As expected, the majority of the Cu-containing proteins were localized in cytoplasm for general metabolism-related enzymes. Because of its function in the electron-transfer chain, some copper proteins were localized at the mitochondrion.

### Iron proteins

**Figure 3:**
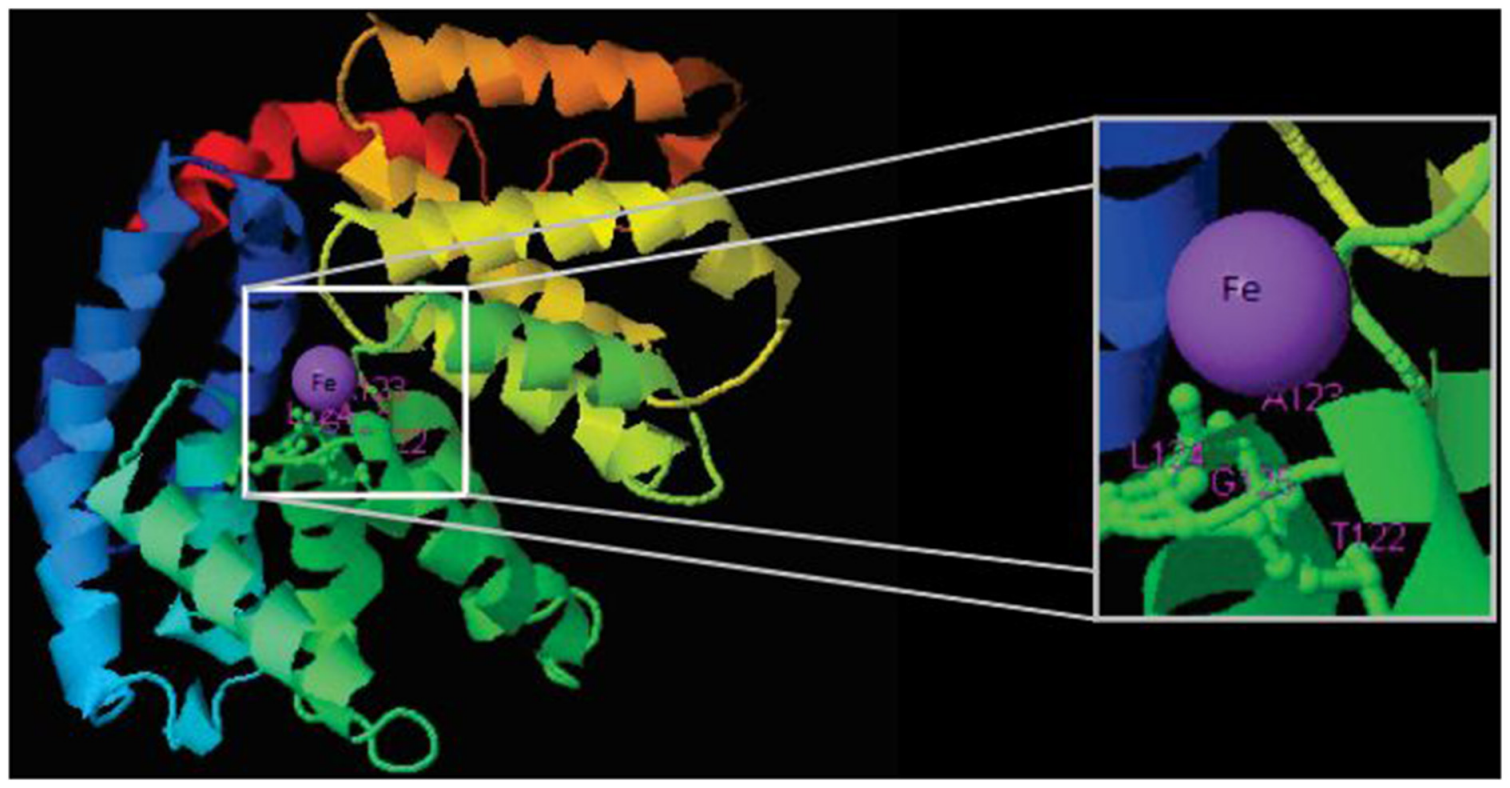
**Structural model of a calcium transporter showing that Fe is the putative ligand**. Structural model was obtained by using the I-Tasser software.

**Figure 4:**
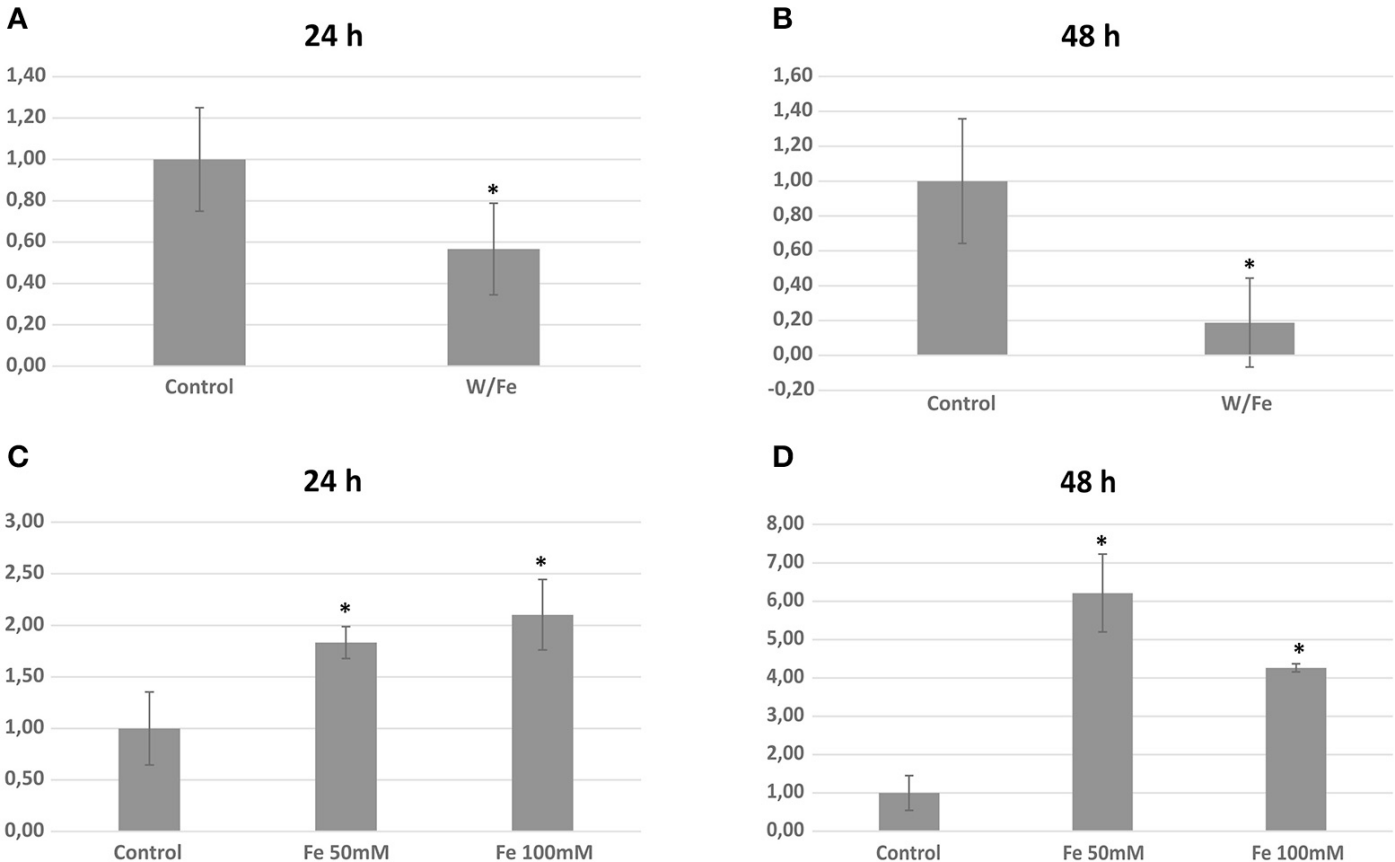
**Expression analysis of the calcium (iron) transporter (PAAG_07762) during iron limitation and iron excess**. Relative transcript level of the transporter in iron limiting condition during 24 h **(A)** and 48 h **(B)**. Relative transcript level of the transporter in iron excess during 24 h **(C)** and 48 h **(D)** ^*^statistically significant differences with *p* < 0.05.

The three genomes encode for 115, 111, and 115 proteins in Pb01, Pb03, and P18, respectively. The general cell metabolism functions were the most enriched category; the primary reason is explained by the presence of many dehydrogenases that catalyze oxide-reduction reactions that use iron to promote electron-transfer processes. Of special note, the metabolism of amino acids and vitamins were highly represented inside this class. Additionally, many protein phosphatases were associated to iron, as expected. Iron proteins related to TCA and electron transfer chains that use iron-sulfur clusters that are essential in aerobic energy production were also found (Figure 1). Accordingly, iron-sulfur cluster assembling proteins compose the iron-proteome of the genus *Paracoccidioides*. Intriguingly, no iron transporter was found using *in silico* analysis. Actually, a calcium transporter (PAAG_07762; PABG_00362; PADG_02775) was found and, consequently, the 3D modeling was built to solve this ambiguity. The analysis showed that this transporter presents iron as a ligand (Figure 3) suggesting that this transporter could act in iron homeostasis in this fungus because no specific iron transporters were found in the genomes available (Silva et al., 2011). To confirm that, expression levels of this transporter were analyzed in conditions with low iron availability and with iron excess. The transcript levels encoding the transporter were decreased in iron deprivation and increased in iron excess suggesting this protein is probably related to iron detoxification more than iron uptake (Figure 4). A urease containing iron as a ligand was also found. However, most of the urease structures available in the PDB are Ni-dependent proteins and only two ureases from bacteria are iron dependent (Carter et al., 2011). *Paracoccidioides* spp. ureases present a higher identity with iron-binding enzymes (57%; PDB ID 3QGA) than with Ni-dependent enzymes (41%; PDB ID 1E9Y; data not shown). Among the iron-binding proteins, three superoxide dismutases were detected.

Most of the iron proteins were predicted to localize in the mitochondrion and cytoplasm. This observation corroborates with the cell processes performed by such molecules. The energy production and electron transfer reactions classically occur in the mitochondrion of eukaryotes. Additionally, proteins related to general metabolic events (amino acid metabolism, carbohydrate metabolism) are predicted to localize in cytosol. Moreover, the plasma membrane and nucleus are cell organelles that contain iron proteins.

### Zinc proteins

Prediction of the *Paracoccidioides* spp. Zn proteomes revealed that zinc proteins are the most abundant metalloproteins encoded by their genome in comparison to Fe and Cu proteins. More than 500 zinc-binding proteins were found to be encoded for each genome (Supplementary Table 1). The most enriched functional categories in the Zn proteome were those related to transcription, cell cycle, and DNA processing, as well as protein fate and modification. Many zinc finger domain containing transcription factors were identified, which contributed to the functional enrichment. Regarding protein fate and modification, many peptidases and proteases were found to be zinc dependent. Additionally, proteins related to phosphorylation and ubiquitination were abundant in this category (Figure 1). Unlike the Cu- and Fe-proteomic data, several metal transporters compose the predicted zinc proteome of the complex *Paracoccidioides* spp. Accordingly, zinc-, metal specific- (other than zinc), heavy metal-, and cation-transporters were found to be zinc binding molecules. A considerable portion of the Zn proteome did not present describe function, reinforcing the fact that the role of metals in several cellular events is beyond the current knowledge. Many Zn proteins are related to nucleic acid related processes and metabolism. Consequently, the nucleus localization is the most frequent protein target in the zinc proteome. Additionally, cytoplasm and mitochondrion localization was also frequent. Corroborating with the high number of metal-transporters, the predicted localization of proteins at the plasma membrane is expressive.

### Comparison of predicted metalloproteomes of three isolates from the *Paracoccidioides* complex

**Figure 5:**
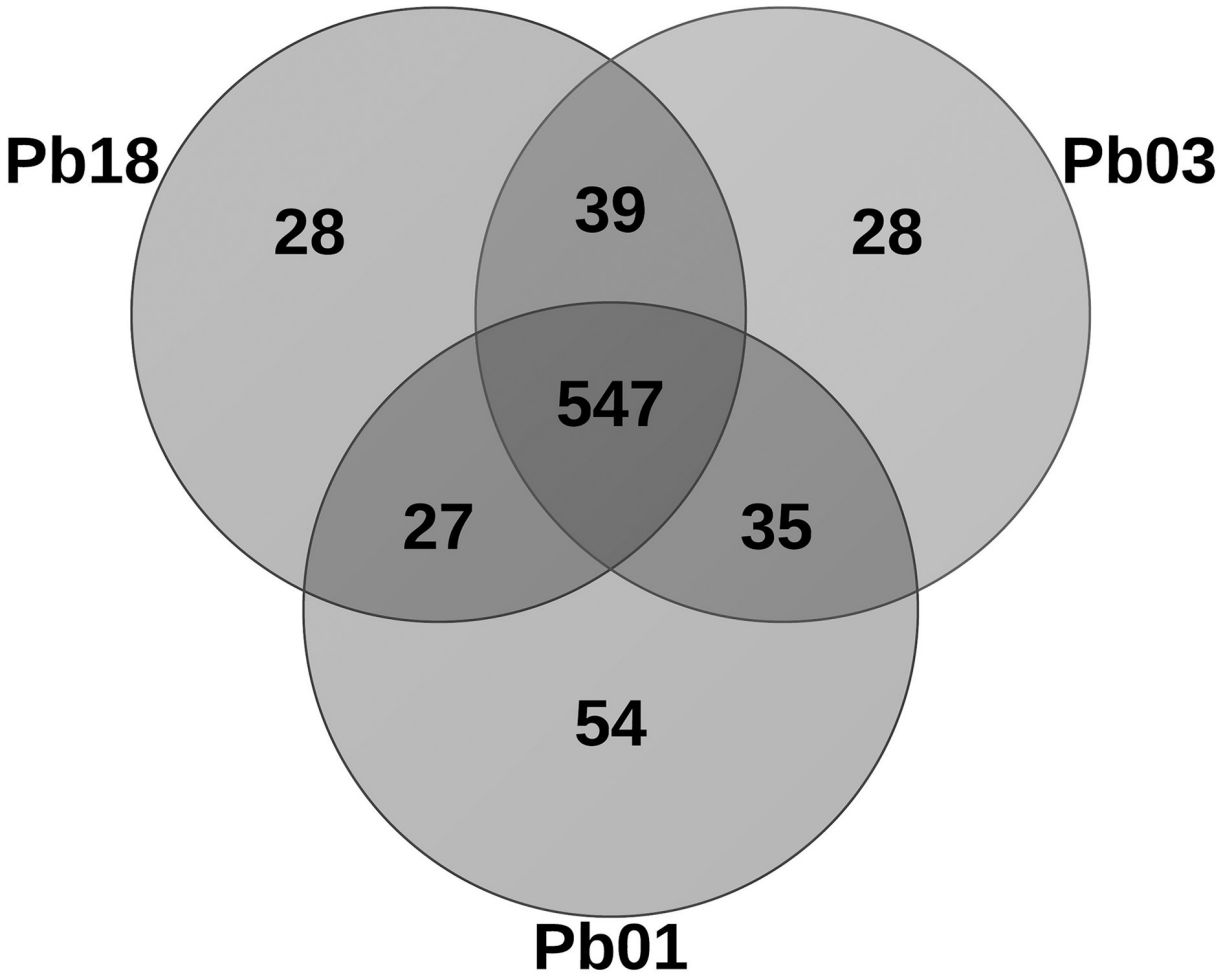
**Venn diagram showing the homology comparisons of metalloproteins found in the three species of *Paracoccidioides* spp**. The numbers represent the how many proteins are specific or common among the species. The comparison was performed using the Blast tool.

The metalloprotein datasets were compared among the three phylogenetic species (PS) by using the BLAST tool. As expected, the comparison showed that most of the proteins are common among the species. Additionally, the specific proteins of each species were identified as well as the specific proteins for two species (Figure 5). *Paracoccidioides lutzii* presented the highest number of specific metalloproteins, which corroborates with the fact that it belongs to a new species in the genus and it has the greater genome. The metal-binding molecules specific to one or two PSs may be a result of different situations: the encoding gene is specific or the encoding gene is not specific but the metal-binding motif is. In the latter group orthologs with no metal-binding domain or orthologs that bind to a different metal were identified, as revealed by the structural prediction.

A comparison of the copper-proteins revealed that each isolate has one exclusive protein and the other five molecules are specific for two isolates according to the BLAST analysis. The exclusive proteins are not encoded by exclusive genes: the protein from Pb01 is not exclusive (orthologs with a Cu-binding domain were found) and the proteins from Pb03 and Pb18 presented orthologs with no metal binding domain (Supplementary Table 5). Regarding the iron proteome, the results revealed that most of the proteins (102 orthologs) are present in the three phylogenetic species. The Pb18 and Pb01 presented six iron-binding molecules that were not found in Pb03. However, Pb03 and Pb18 genomes encode five specific orthologs, whereas Pb01 and Pb03 have 3 iron proteins not found in Pb18. Although the Pb18 iron proteome has no specific protein, Pb01 and P03 present three specific proteins and one specific protein, respectively. None of these proteins are encoded by specific genes. The BLAST search coupled to structural prediction analysis revealed three different situations: a specific iron protein (there are orthologs in other phylogenetic species but a metal-binding protein was not found in one or two species); the iron proteins are not specific (a structural I-Tasser analysis developed iron-dependent models in other species); or the protein presents many orthologs in the other species that an arbitrary selection could not drive a reliable conclusion. The comparison of the zinc-binding proteomes of three genomes shows that a high portion is conserved among them (Supplementary Table 5). Regarding Pb01 and Pb03, most of the specific Zn proteins are not encoded by specific genes and, therefore, there are peptides with Zn-binding domains specific to a single phylogenetic species. Proteins encoded by species-specific genes were also found, as well as, proteins that are not specific.

### Metal dependent virulence factors

Metal essentiality and toxicity is used by a host to defend against invaders, thus metal uptake and homeostasis by pathogens in mammal host tissues are essential for virulence (Hood and Skaar, 2012). Some metalloproteins are essential for the intracellular regulation of metals, as well as for the control of their import and export (Sun et al., 2011). Pathogens must acquire and control metal utilization during infection to survive in their hosts (Silva et al., 2011). Several metalloproteins identified in this work play roles in fungal pathogenesis (Table 3). Classical virulence factors compose the predicted metalloproteomes in *Paracoccidioides* spp. For example, five superoxide dismutases were found: two are Cu and Zn-dependent and three are Fe-dependent. Thus, these metals become essential elements to an effective response against ROS production by the host. Laccase enzymes are related to melanin production and iron uptake, which contributes to the virulence of pathogenic fungi present in copper-binding domains. Urease is another enzyme that contributes primarily to fungal pathogenesis and was identified as an iron protein. This protein promotes changes in pH and toxic effects to increase the fungal pathogenicity (Mirbod-Donovan et al., 2006). Alcohol dehydrogenases (ADH) are related to the *Paracoccidioides* spp. response to host mimicking conditions. Five ADH genes were found to be zinc-binding molecules and one as iron-binding. Additionally, other Zn-binding enzymes related to central carbon metabolism such as fructose 1,6-biphosphate aldolase, triose phosphate isomerase and enolase have moonlight functions related to the adhesion to host cells and tissue dissemination. Protease production is a strategy used by several pathogens in host colonization and many proteases are metal-dependent proteins as found in our analysis. Also we found that many peptidases and proteases were related to protein processing rather than virulence. As metals are essential to the molecules of live organisms, the components that control their uptake and utilization have been described as factors that contribute to virulence. Accordingly, several cation/metal transporters were identified in the zinc metalloproteome of the genus *Paracoccidioides*. Among several zinc-dependent transcription factors the GATA-type Cir1/SREA (PAAG_00610, PABG_05322, PADG_06931/PAAG_02358, and PABG_04857) related to iron homeostasis and virulence in fungi was found. Previous analysis suggested that *Paracoccidioides* spp. are devoid of a classical high affinity iron transport Ftr (Silva et al., 2011). In the present work, a putatively iron-transporter that contributes to the virulence of *C. albicans* and *C. neoformans* was found (Ramanan and Wang, 2000; Jung et al., 2008). Additionally, copper homeostasis plays an essential role in fungal pathogenesis. High affinity copper transporters (CTR) are key elements in copper uptake in Cu-limiting conditions (Waterman et al., 2007, 2012). The CTR3 of *Paracoccidioides* spp. was identified in our analysis and its transcripts are induced during infectious mimicking conditions (Bailao et al., 2006; Costa et al., 2007). Other copper transporters (copper-transporting ATPases) are localized at the Golgi complex and a copper-chaperone participates in melanin synthesis in *C. neoformans*, suggesting their role in virulence (Walton et al., 2005).

**Table 3:** **Metalloproteins described as virulence factors in fungi**.

**Pb01 access number**	**Pb03 access number**	**Pb18 access number**	**Protein description**	**Virulence factor's reference**	**Metal bound**
PAAG_00610	PABG_05322	PADG_06931	GATA transcription factor	Hwang et al., 2012	Zn
PAAG_02358	PABG_04857	PADG_05497	GATA factor SREP	Hwang et al., 2012	Zn
PAAG_04164	PABG_03954	PADG_07418	superoxide dismutase	Cox et al., 2003	Zn
PAAG_02971	PABG_00431	PADG_02842	cytosolic Cu/Zn superoxide dismutase	Cox et al., 2003	Cu/Zn
PAAG_06363	PABG_02770	PADG_01263	superoxide dismutase	Cox et al., 2003	Fe
PAAG_02725	PABG_03204	PADG_01755	superoxide dismutase	Cox et al., 2003	Fe
PAAG_02926	PABG_03387	PADG_01954	superoxide dismutase	Cox et al., 2003	Fe
PAAG_03681	PABG_00738	PADG_03184	laccase-1	Zhu et al., 2001	Cu
PAAG_06004	PABG_05667	PADG_05994	laccase-IV	Zhu et al., 2001	Cu
PAAG_00163	PABG_05183	PADG_07092	laccase-3	Zhu et al., 2001	Cu
PAAG_00954	PABG_01291	PADG_03871	urease	Mirbod-Donovan et al., 2006	Fe
PAAG_00243	None	None	alcohol dehydrogenase IV	Pancholi and Chhatwal, 2003	Fe
PAAG_08903	PABG_05423	PADG_05734	alcohol dehydrogenase	Pancholi and Chhatwal, 2003	Zn
PAAG_06916	PABG_02939	PADG_01454	alcohol dehydrogenase	Pancholi and Chhatwal, 2003	Zn
PAAG_06596	PABG_02619	None	alcohol dehydrogenase	Pancholi and Chhatwal, 2003	Zn
PAAG_06715	PABG_02727	PADG_01174	alcohol dehydrogenase	Pancholi and Chhatwal, 2003	Zn
PAAG_05227	PABG_07631	PADG_05031	alcohol dehydrogenase	Pancholi and Chhatwal, 2003	Zn
PAAG_04541	PABG_04316	PADG_04701	alcohol dehydrogenase GroES domain-containing protein	Pancholi and Chhatwal, 2003	Zn
PAAG_06104	PABG_06552	PADG_08012	fructose-biphosphate aldolase	Pancholi and Chhatwal, 2003	Zn
PAAG_01995	PABG_02260	PADG_00668	fructose-biphosphate aldolase	Pancholi and Chhatwal, 2003	Zn
PAAG_02152	PABG_02388	PADG_00743	Class II aldolase family protein	Pancholi and Chhatwal, 2003	Zn
PAAG_00557	PABG_03558	PADG_02132	mannose-6-phosphate isomerase	Pancholi and Chhatwal, 2003	Zn
PAAG_00771	PABG_01457	PADG_04059	enolase	Pancholi and Chhatwal, 2003	Zn
PAAG_07076	PABG_03073	PADG_01601	M6 family metalloprotease	Puccia et al., 1999	Zn
PAAG_05251	None	None	High affinity copper transporter	Bailao et al., 2006; Waterman et al., 2012	Cu
PAAG_07053	PABG_03057	PADG_01582	Copper-transporting ATPase	Walton et al., 2005	Cu
PAAG_07154	PABG_02495	PADG_00917	Copper-transporting ATPase	Walton et al., 2005	Cu
PAAG_07762	PABG_00362	PADG_02775	Calcium transporter (iron transporter)	Ramanan and Wang, 2000; Jung et al., 2008; Silva et al., 2011	Fe

## Discussion

The essentiality of metals in biology, such as Cu, Fe, and Zn, is most related to metal-dependent proteins that play fundamental roles in cells. However, the intracellular levels of these elements have to be tightly controlled because their excess is toxic (Yannone et al., 2012). Regarding pathogenic microorganisms, the uptake, storage, use and distribution of metals are key factors for virulence. To establish infection, fungal pathogens must acquire and use metals in host tissues to cope with the metal scarcity induced by the immune system (Vignesh et al., 2013a,b). In this perspective the pathogen also has to adapt to a low level metal environment based on its metalloproteome, changing its metabolism, as much as possible, toward metal independent pathways (Parente et al., 2011).

Not unexpectedly, copper protein functions are most related to ion transport, virulence and general metabolism. The latter include metabolic oxidases such as amine oxidases, glyoxal oxidase, laccases and other oxidases that use the copper redox ability in oxidoreduction/electron-transfer reactions. The dependence of copper by amine oxidases and nitrite reductases suggests that nitrogen metabolism of these fungi is supported by Cu (Laliberte and Labbe, 2006). The *Paracoccidioides* spp. Cu proteomes present the primary molecular components of copper uptake and distribution. Among those found, the copper-transporting ATPases are important in metal detoxification and trafficking, as well as, in delivering metal to copper proteins. The laccases are ferroxidases, essential in the reductive iron uptake system and in melanin synthesis, were also found. Other factors important in copper homeostasis are the high affinity Cu-transporting and copper chaperones (Festa and Thiele, 2011, 2012). Additionally, the functional interaction network for Cu-protein suggests a clear linkage among those that orchestrate several Cu-dependent cellular mechanisms, including activities that sustain pathogenesis. It is important to highlight the strong functional association among copper-chaperones, copper-transporting ATPases, and laccases that shows the central roles of those proteins in cellular copper equilibrium. The functional interactions among these proteins are highly confident, suggesting that Cu acquisition and distribution in the fungal cells are tightly controlled processes preventing increases in free-copper levels.

The analysis of the functional repertoire performed by the putative Fe proteins revealed that the primary roles played by the Fe proteins are the catalysis of redox and electron transfer reactions. At a relatively general level, metabolism is the most representative functional category; it is composed of proteins that belong to amino acid metabolism, biosynthesis of vitamins, nucleotide metabolism, carbohydrate metabolism and others. The amplitude of these metabolic processes is evidence as to how important iron is for basal fungal metabolism. Corroborating that importance, a proteomic analysis in iron starvation condition suggests *Paracoccidioides* spp. decrease iron dependent enzymes and consequently increase proteins of iron independent pathways (Parente et al., 2011).

Previous work, based on genome-mining, shows that *Paracoccidioides* spp. genomes encode proteins related to the reductive iron uptake system (Silva et al., 2011). However, no iron transporter was found in this study, and it was assumed that the iron uptake would be performed by a non-specific metal transporter. In the present work, among the iron proteins is a predicted iron-transporter formerly annotated as a calcium transporter. Structural modeling of this transporter suggested that iron is the metal ligand. The quantitative PCR results show that the expression of this transporter is regulated by iron and strongly suggests that it plays a role in metal detoxification rather than in uptake. Moreover, this ambiguity is common in metalloproteome studies because protein metal affinity is a multifactorial event and wrong annotations or structures are frequently detected (Maret, 2010). Future functional studies should be conducted to better elucidate the specific role of this molecule in iron homeostasis. It is well established that defects in iron-sulfur cluster biogenesis or transport induce the expression of iron uptake genes (Chen et al., 2004) and that Fe-S containing proteins participate in sensing iron availability (Muhlenhoff et al., 2010). The presence of many iron-sulfur cluster assembly proteins in the predicted iron proteome described here provides new molecules that may perform roles in Fe homeostasis.

The Zn-proteome of *Paracoccidioides* spp. represents the majority of the metalloproteins described in the present work. 511 proteins were identified with at least one metal-binding motif, on average. The number of putative zinc proteins found in each species directly correlates with their predicted proteome sizes. As expected, proteins related to gene expression were the most abundant (20% on average) because transcription factors use zinc for structural reasons to bind to DNA. Thus, this enrichment is responsible for the high frequency of Zn proteins found in eukaryotes (Andreini et al., 2006b). Among them is the GATA-type transcription factor SREP (accession numbers PAAG_02358, PABG_04857, and PADG_05497), which inhibits the expression of iron-uptake related genes in iron-sufficient conditions, and also regulates some iron-independent genes. Additionally, SREP mutants presented abrogated virulence in pathogenic fungi (Gauthier et al., 2010; Hwang et al., 2012). The most populated functional categories include protein folding, processing and degradation. Additionally, some protein importers, kinases/phosphatases and many proteases and peptidases are zinc dependent. Regarding pathogens, the protease activity is intimately connected with virulence (Parente et al., 2005, 2010; Tacco et al., 2009). Thus, zinc homeostasis during infection supports protease based virulence, as well as protein transport and processing in the genus *Paracoccidioides*.

The Zn proteome of *Paracoccidioides* spp. presents at least 17 transporters related to Zn translocation. The *Paracoccidioides* spp. Zinc proteome contains many metal transporters including Zrt1 and Zrt2, which are localized at the plasma membrane and are related to metal uptake during Zn-limiting and Zn-replete conditions, respectively. The expression of both genes is induced at the fungal cells (Parente et al., 2013). Vacuolar transporters that regulate the cytoplasmic levels of the metal were also identified (Amich et al., 2010). A metallochaperone previously described as a copper-binding protein was identified in this study as a Zn-binding molecule. This is not surprising as the same has been observed in bacterial chaperones (Dainty et al., 2010). Considering *Paracoccidioides* spp. have no predicted metallothionein (Silva et al., 2011), this molecule could somehow play a role in metal level maintenance.

Recently, a metallomics-based study unveiled that the host immune response against a fungal pathogen promotes zinc limitation in fungal-infected cells. GM-CSF treated macrophages promote zinc uptake to generate ROS as a fungicidal mechanism, and at the same time decrease Zn availability by metallothionein production and zinc Golgi sequestration (Vignesh et al., 2013a). Thus, metalloproteins that allow *Paracoccidioides* spp. to uptake and distribute zinc during infection is an essential event for survival in host niches. *Paracoccidioides* spp. induce the production of SODs during oxidative stress, as demonstrated by the proteomic approach (De Arruda Grossklaus et al., 2013). These enzymes are metal dependent and are classified in accordance to their metal as Cu/ZnSOD, MnSOD, FeSOD, and NiSOD (Broering et al., 2013). *Paracoccidioides* spp. encode for five different SODs: two are copper- and zinc-binding proteins and the other three are Fe-SODs. As these metalloenzymes are important components in the arsenal of fungal virulence factors, an efficient combat against host microbicide ROS rely on a virtuous copper, iron and zinc uptake and homeostasis during infection. Melanization is a virulence factor of many pathogenic fungi (Nosanchuk and Casadevall, 2006; Taborda et al., 2008). *Paracoccidioides* spp present one tyrosinase and three putative laccases that are key copper-oxidase enzymes in melanin synthesis. Furthermore, metallochaperones and copper transporters play a critical role on melanin production as both proteins participate in Cu-loading in laccases (Festa and Thiele, 2012). The urease production contributes to the virulence of microorganisms (Rutherford, 2014). Although nickel is the most common metal found in urease structures, an iron-binding urease was found in the present analysis. Moreover, as no fungal urease structure is available, these data can be used in future structural studies. Additionally, the enolase, a Zn-binding protein of these pathogens, interacts with host plasminogen favoring the invasion and dissemination steps during host tissue infection (Nogueira et al., 2010).

## Conclusion

A previous study described the association of zinc metabolism and fungal virulence based on the identification of zinc-binding proteins and a literature review. The authors used an annotation based approach only, which is not a first line technique for identifying metalloproteins in databases (Andreini et al., 2006b). The present work was used a systematic bioinformatic approach based on domains and conserved structures available in the PDB to predict the Cu, Fe, and Zn proteomes of a pathogenic fungus. The results show that these metals are cofactors of enzymes related to central metabolism and thus are essential for the biology of *Paracoccidioides* spp. It was also noted that many metalloproteins have no characterized function, indicating that the roles of many metals in biological systems are still unknown. Additionally, some metalloproteins belong to the arsenal of virulence factors taken by *Paracoccidioides* spp. to be able to infect hosts. Although bioinformatics based studies are important to identify metalloproteins, experimental techniques will be used to characterize the metalloproteins of these pathogenic fungi.

## Author contributions

Gabriel B. Tristão, Mirelle Garcia Silva-Bailão, Clayton Luiz Borges, Gabriele Cavallaro and Leandro do Prado Assunção performed the analysis, data analysis and writing. The following contributed to financial support: Alexandre M. Bailão, Gabriele Cavallaro, Célia M. de Almeida Soares and Clayton Luiz Borges. Leandro do Prado Assunção and Luiz Paulo Araújo dos Santos analyzed the metalloproteome data. Alexandre M. Bailão and Gabriele Cavallaro conceived the ideas, performed the experimental design and wrote the paper.

### Conflict of interest statement

The authors declare that the research was conducted in the absence of any commercial or financial relationships that could be construed as a potential conflict of interest.
